# 405 nm violet-blue light inactivates hepatitis C cell culture virus (HCVcc) in ex vivo human platelet concentrates and plasma

**DOI:** 10.1038/s41598-024-83171-3

**Published:** 2024-12-28

**Authors:** Joseph W. Jackson, Pravin R. Kaldhone, Caitlin Stewart, John Anderson, Scott MacGregor, Michelle Maclean, Marian Major, Chintamani D. Atreya

**Affiliations:** 1 Division of Blood Components and Devices, Center for Biologics Evaluation and Research, FDA, Silver Spring, MD 20993 USA; 2 Department of Electronic and Electrical Engineering, The Robertson Trust Laboratory for Electronic Sterilization Technologies (ROLEST), University of Strathclyde, Glasgow, UK; 3 Department of Biomedical Engineering, University of Strathclyde, Glasgow, UK; 4 Division of Viral Products, Center for Biologics Evaluation and Research, FDA, Silver Spring, MD 20993 USA; 5 Present Address: Congressional Research Service, Library of Congress, Washington, DC 20540 USA

**Keywords:** Pathogen inactivation, HCV, Platelets, Plasma, blue light, 405 nm light, Pathogens, Biophotonics

## Abstract

Added safety measures coupled with the development and use of pathogen reduction technologies (PRT) significantly reduces the risk of transfusion-transmitted infections (TTIs) from blood products. Current approved PRTs utilize chemical and/or UV-light based inactivation methods. While the effectiveness of these PRTs in reducing pathogens are well documented, these can cause tolerable yet unintended consequences on the quality and efficacy of the transfusion products. As an alternative to UV-based approaches, we have previously demonstrated that 405 nm violet-blue light exposure successfully inactivates a variety of pathogens, including bacteria, parasites, and viruses, in both platelet concentrates (PCs) and plasma. Herein, we show that 405 nm light treatment effectively inactivates hepatitis C cell culture virus (HCVcc) by up to ~ 3.8 log10 in small volumes of a variety of matrices, such as cell culture media, PBS, plasma, and PCs with 27 J/cm^2^ of light exposure, and total inactivation of HCVcc after 162 J/cm^2^ light exposure. Furthermore, we demonstrate that carry-over of media supplemented with fetal bovine serum enhances the production of reactive oxygen species (ROS), providing mechanistic insights to 405 nm light-mediated viral inactivation. Overall, 405 nm light successfully inactivates HCVcc, further strengthening this method as a novel PRT for platelets and plasma.

## Introduction

In transfusion medicine, threats from known and unknown transfusion-transmitted infections (TTIs) pose a serious risk to the safety of the public health. The most common transfusion products are ex vivo blood components (plasma, platelet concentrates and packed red blood cells) stored in blood banks and hospitals for patient care. There is a risk of infection transmission via blood components. Although the risk is reduced by implementation of several layers of safety, the residual risk is unavoidable^1^. To mitigate the residual risk, the field has been proactively developing and implementing robust pathogen inactivation (PI) treatments through the application of pathogen reduction technologies (PRTs) for whole blood and blood component safety^2,3^. Current PRTs utilize chemical and/or UV light-based inactivation methods to reduce pathogen burden in blood components. Whilst being effective in reducing either pathogen levels or their infectivity, it is well documented that these technologies cause tolerable, unintended consequences on the quality and efficacy of transfusion products particularly with regard to UV light exposure^4,5^.

To mitigate the effects of UV light on the products, our research focus has been on utilizing high intensity narrow spectrum (HINS) 405 nm violet-blue light that falls within the visible light spectrum as an alternative to UV light. Previously, we successfully demonstrated its potential as a pathogen inactivation or reduction tool for human plasma and platelets stored in plasma, by experimentally contaminating the two blood components with a number of bacteria, blood-borne protozoan parasites (*Trypanosoma cruzi* and *Leishmania donovani*) and viruses such as feline calicivirus (FCV) and human immunodeficiency virus (HIV-1)^6–12^. We have also demonstrated that the violet-blue visible light-treated platelets behave similar to the light-untreated platelets in vivo in a severe combined immunodeficient (SCID) mouse model, and that there are neither visible changes to plasma protein integrity nor protein oxidation in human plasma treated with 405 nm light doses that completely inactivate bacteria and protozoan parasites^13,14^. Further, we also recently demonstrated that the light treated platelet concentrates (PCs) retain hemostasis activity which is an essential function of the platelets stored for transfusion and the light treatment show little to no negative effect on plasma quality^15,16^.

In this report, 405 nm light was evaluated on human PCs and plasma spiked with hepatitis C cell culture virus (HCVcc) to determine whether this enveloped virus can be inactivated. Hepatitis C virus (HCV) is a global public health burden with ~ 50 million individuals chronically infected worldwide^17^. Exposure to blood and blood products is one of the major sources of infection with the majority of cases (70%) becoming chronic, which can result in liver damage and hepatocellular carcinoma^18^. Although direct-acting antiviral drugs (DAAs) achieve high rates of clearance in infected individuals (> 99%), DAA treatment is limited due to cost and access to care and clearance with DAAs does not prevent new infections occurring in the same patient that can again become chronic^19,20^. There is currently no vaccine against HCV, therefore, prevention of transmission remains one of the most important approaches to reducing incidence rates. Although transmission through blood transfusions has become relatively rare, nosocomial transmission still accounts for a large proportion of new infections^21^. HCV can remain infectious on surfaces for extended periods of time^22,23^ and the development of new methods that can inactivate the virus on or within different systems can contribute significantly to preventing all forms of transmission. Natural isolates of HCV cannot be grown in cell culture, however, in 2005 a single isolate was adapted to grow in human hepatoma cells^24^ and this virus (termed HCV cell culture virus, HCVcc) represents a useful tool to perform infectivity studies in vitro. The results demonstrate that inactivation of HCVcc in human plasma is successful with the violet-blue light treatment. This observation, together with our previously demonstrated proof-of-concept studies on the pathogen inactivation potential of 405 nm light, reinforces our vision that 405 nm light can be harnessed as a broad-spectrum pathogen reduction technology for further evaluation towards safety of the blood components from TTIs.

## Materials and methods

### Human platelets, plasma, and HCVcc and HCV-related material

Human apheresis platelet concentrates (PCs) stored in plasma from individual donors (*n* = 6), were received from the National Institutes of Health (NIH) Blood Bank, Department of Transfusion Medicine (Bethesda, MD, USA) and stored at 22 °C under gentle agitation. All experiments involved PCs and plasma that were stored for 1–3 days. The study involving human subjects’ protocol was approved by FDA Research Involving Human Subjects Committee (RIHSC. Exemption Approval #11-036B), and all methods were performed in accordance with the relevant guidelines and regulations. We used both PCs and plasma derived from PCs *aka* Platelet-poor-plasma (PPP) in our experiments. The latter was prepared from each unit of PC by centrifugation at 4,000x*g* for 30 min using horizontal swing buckets and supernatant was carefully collected and labeled as plasma.

J6/JFH1 hepatitis C cell culture virus (HCVcc) was used in these experiments. The virus stock was prepared by transfecting full-length HCV RNA derived from an HCV genotype 2a clone (a gift from Charles Rice, Rockefeller University, New York, NY) into Huh 7.5 cells as previously described^25^. HCV pseudotyped particles carrying the envelope E1E2 of GT1a (H77, AF009606) (HCVpp) and a non-enveloped control (No Envpp) were generated as previously described^26^. Soluble HCV E2 glycoprotein representing amino acids 384–661 of the HCV polyprotein (H77, AF009606) was purified from the supernatant of Expi293F cells as previously described^27^. For evaluation of virucidal efficacy of 405 nm light on HCVcc in different liquid matrices, we spiked the virus in small volumes (0.3–1.0 mL) of PBS, Dulbecco’s Modified Eagle Medium (DMEM), DMEM supplemented with Riboflavin (0.4 µg/mL), plasma and PCs (platelets stored in plasma).

### 405 nm light exposure

**Fig. 1:**
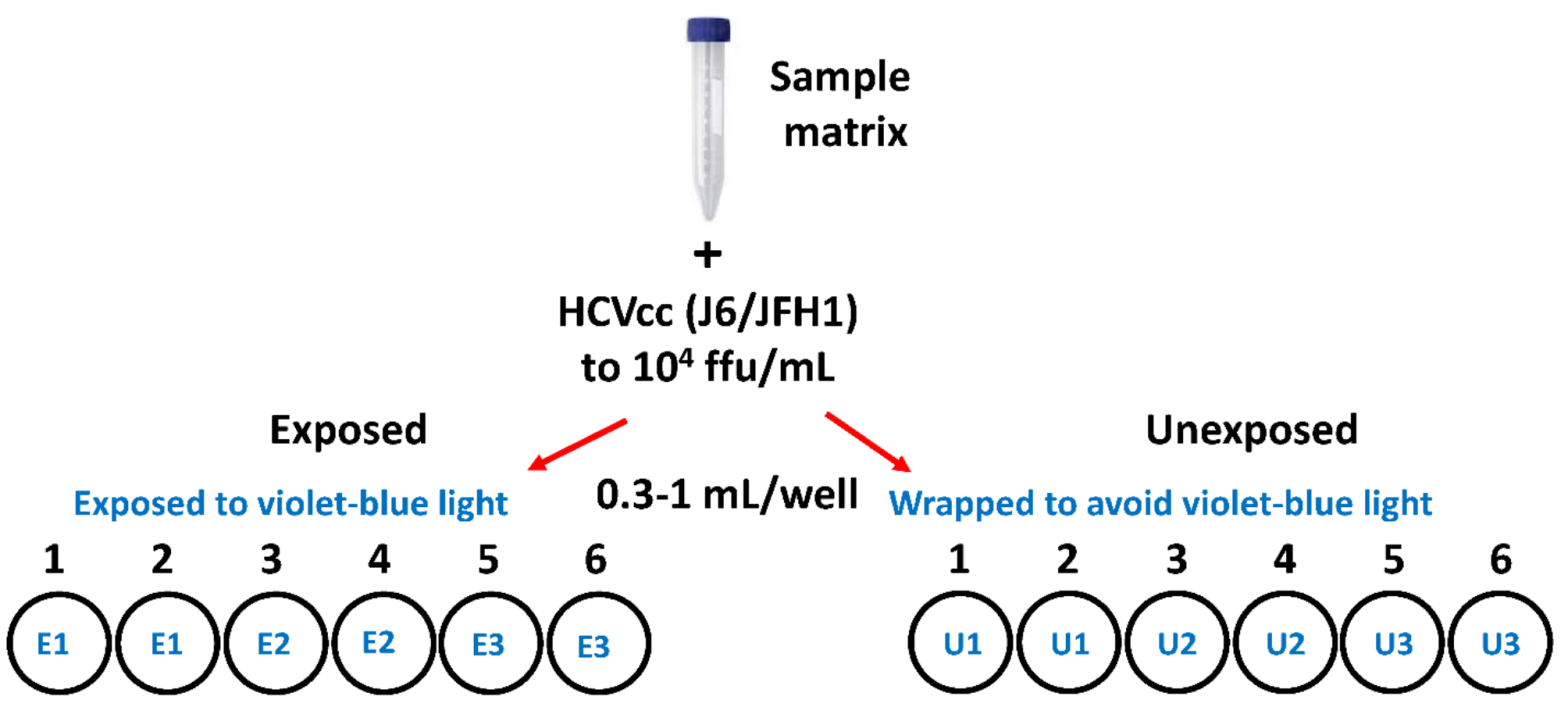
Study design for HCVcc inactivation. Specific matrices were spiked with HCVcc to a titer of 10^4^ ffu/mL. Spiked samples (0.3 ml or 1 mL) were transferred in duplicate to 12 or 48 well plates. One plate was exposed to 405 nm violet-blue light (Exposed), the 2nd plate was wrapped in a light impenetrable cover and exposed to 405 nm violet-blue light (Unexposed). At specific time points samples were removed and tested for virus titers.

Two 48 well or 12 well plates were used for each experiment. One was designated as the “Exposed” plate and the other as the “Unexposed” control, which was wrapped in a light impenetrable cover (Fig. 1). Sample matrices were spiked with HCVcc (to a titer of 10^4^ focus forming units (ffu)/mL) and 0.3 mL (48 well plate) or 1 mL (12 well plate) of spiked sample was transferred to wells in each of the exposed and unexposed plates (Fig. 1). Two replicates of a sample were placed for each timepoint. Both plates were incubated in a closed system that delivers 405 nm light at 22 ºC with shaking at 60 rpm. In this closed light system, each 0.5 h of exposure delivers 27 J/cm^2^ of 405 nm light dose. The samples were subjected to a time course of 405 nm light exposure varying from 0.5 h (27 J/cm^2^) to 5 h (270 J/cm^2^). At specified time points, duplicate samples were removed from the exposed and unexposed plates and virus titers were immediately assessed on Huh7.5 cells.

### Assessment of HCVcc titers in spiked samples

To assess virus titers, samples (undiluted and diluted 1:10) in complete growth medium (DMEM supplemented with 10% FBS/1% penicillin/streptomycin/2 mM glutamine/non-essential amino acids) were inoculated onto Huh7.5 cells, 50 µL per well in duplicate. After incubation at 37 °C/5% CO_2_ for 3 h, samples were removed and replaced with fresh complete growth medium. Cells were incubated for 3 days at 37 °C/5% CO_2_, then fixed, and stained as previously described^25^ using a monoclonal antibody that recognizes the HCV core antigen (6G7). Positive focus forming units (FFU) were counted and titers calculated as FFU/mL. The limit of detection (LOD) for the assay is 20 FFU/mL. Samples with no detectable foci were assigned a titer of half the LOD (10 FFU/mL).

### Fluorogenic assay to detect reactive oxygen species

We determined reactive oxygen species (ROS) in samples using dichlorofluores *cin* diacetate (H_2_DCFDA, catalog number: D399, Thermo Fisher Scientific Inc, Waltham, MA, USA), a fluorogenic indicator of ROS, as previously described^7^. Briefly, 0.3 mL complete DMEM (DMEM supplemented with 10% FBS/1% penicillin/streptomycin/2 mM glutamine/non-essential amino acids), unsupplemented DMEM, HCV pseudotyped particles (HCVpp), a non-enveloped control (No Envpp) (all diluted 1:5 with 1× PBS), and HCV E2 glycoprotein (3 μg/mL in PBS) were added to two 48 well plates (1 designated as Unexposed and 1 designated as Exposed) and subjected to 405 nm light treatment for either 0.5 h (27 J/cm^2^) or 1 h (54 J/cm^2^). All samples were assessed in duplicate. After exposure, the samples were collected and kept on ice while fresh H_2_DCFDA compound was prepared. Subsequently, 0.2 mL of each sample (in duplicate) was dispensed into a 96 well black wall clear bottom plate (Corning, Inc., Corning, NY, USA). PBS alone served as a negative control, while PBS with 0.5 µM hydrogen peroxide (H_2_O_2_) served as the positive control. The H_2_DCFDA reaction was initialized by adding 20 µM H_2_DCFDA to each sample and incubated for 30 min at 37 °C. The resulting fluorescence in relative fluorescence units (RFU) was measured on a spectrophotometer at an excitation/emission of 492/532 (Spectramax iD5, Molecular Devices, San Jose, CA, USA).

### Statistical analyses

All statistical tests were performed in GraphPad Prism software using Mann Whitney test. A p value < 0.05 was considered statistically significant.

## Results

### 405 nm light inactivates HCVcc in DMEM and PBS

**Fig. 2:**
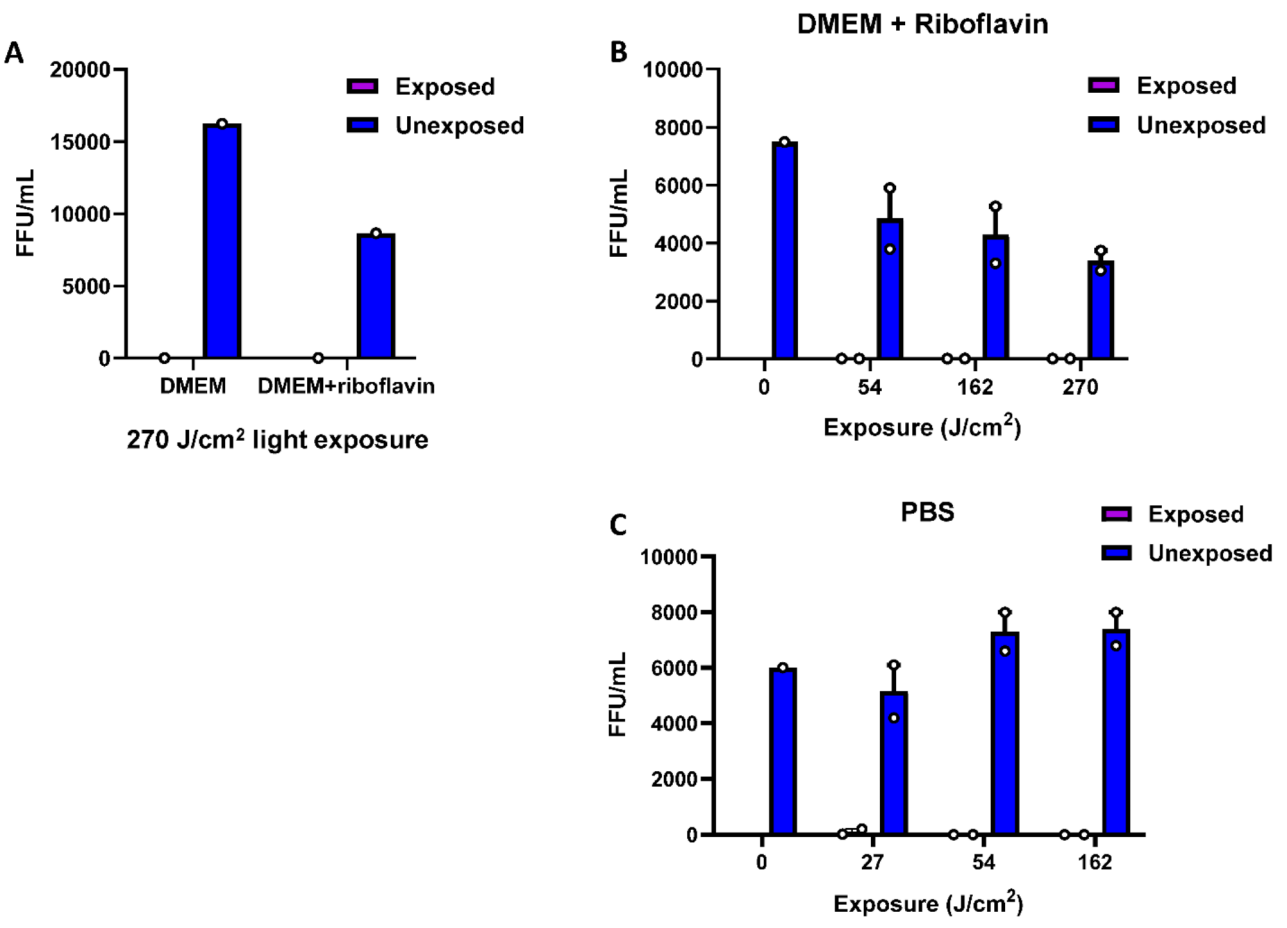
405 nm light inactivates HCVcc in DMEM and PBS. (**A**) HCVcc diluted in DMEM to a titer of 10^4^ ffu/mL, with and without riboflavin (0.4 µg/mL) and exposed to 270 J/cm^2^ 405 nm violet-blue light or exposed after enclosure in a light impenetrable cover (Unexposed). (**B**) Dose response study of HCVcc diluted in DMEM plus riboflavin (0.4 µg/mL) and exposed to 405 nm violet-blue light or exposed after enclosure in a light impenetrable cover (Unexposed). (**C**) Dose response study of HCVcc diluted in PBS and exposed to 405 nm violet-blue light or exposed after enclosure in a light impenetrable cover (Unexposed). Bars represent the mean of duplicate tests; error bars represent standard error. Each dot represents an individual test value. No HCVcc titers were evaluated for exposed at time 0, the value for Unexposed at time 0 represents untreated samples. Note that around at 54 J/cm^2^ light dose, the 10^4^ ffu/mL virus was inactivated to below detection levels, demonstrating approximately a 4log_10_ reduction efficacy.

Preliminary experiments were performed to test the efficiency of HCVcc inactivation using virus spiked DMEM and PBS. Since virus particles (virions) are believed to be devoid of photosensitizers, in our preliminary experiments, we tested DMEM supplemented with riboflavin as one of the matrices. Riboflavin is a photosensitizer useful in UV and visible light-based inactivation of pathogens found in blood products^28^. Several studies on 405 nm light have established that the light excites both internal and external photosensitizers (present in the surrounding media) resulting in radical oxygen species (ROS) generation through which microbicidal activity of 405 nm light is manifested^29^. We found that HCVcc infectivity was completely abrogated in DMEM with or without riboflavin (0.4 µg/mL) after exposure to 270 J/cm^2^ of light (Fig. 2A). Subsequently, in a dose response study, we found that after exposure to 54 J/cm^2^ of light, HCVcc infectivity was no longer detectable in either DMEM supplemented with riboflavin (Fig. 2B) or, in PBS (Fig. 2C), although small amounts of virus were detectable after exposure to only 27 J/cm^2^ of light in PBS (Fig. 2C). Based on these experiments, subsequent experiments were conducted with the light exposures limited to 162 J/cm^2^ and without supplementation of riboflavin.

### 405 nm light inactivates HCVcc in human PCs and plasma

**Fig. 3:**
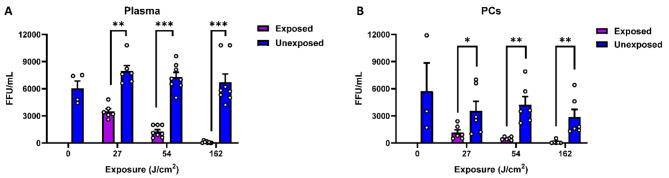
405 nm light inactivates HCVcc in plasma and platelets. (**A**) Plasma samples spiked with HCVcc and exposed to increasing doses of 405 nm violet-blue light. (**B**) Platelet samples spiked with HCVcc and exposed to increasing doses of 405 nm violet-blue light. Bars represent the means of individual donors; error bars represent standard error. Each dot represents an individual donor. No HCVcc titers were evaluated for exposed at time 0, the value for Unexposed at time 0 represents untreated samples. Statistical analysis was conducted using Mann–Whitney test. Significance levels are indicated as follows: *p < 0.05, **p < 0.01, ***p < 0.001.

**Fig. 4:**
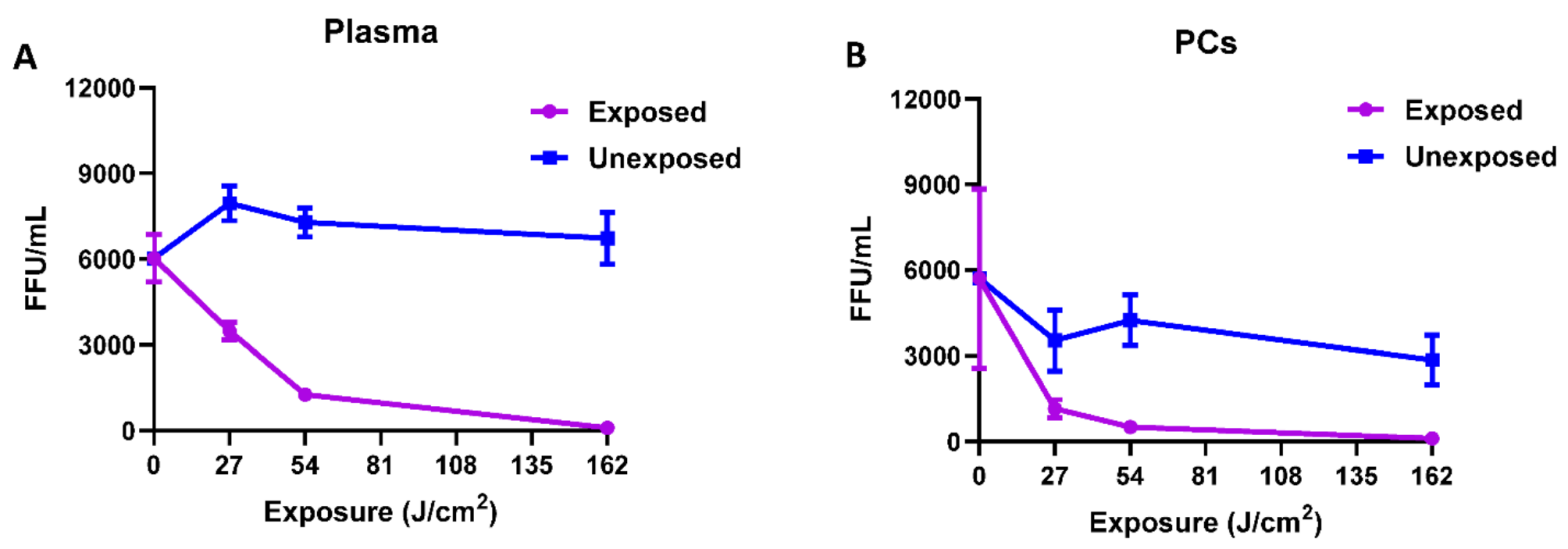
HCVcc inactivation response to 405 nm light dosing. (**A**) Kinetics of HCVcc inactivation in plasma samples. (**B**) Kinetics of HCVcc inactivation in platelets. Values represent the means of multiple donors. Error bars represent standard error.

Using data obtained with DMEM and PBS as the matrices for HCVcc spiking, we performed dose response studies using both plasma and PCs (Figs. 3 and 4). The virus was spiked into donor samples and duplicate samples of each donor were exposed to increasing amounts of light, from 27 to 162 J/cm^2^. As observed for the minimal matrices of DMEM and PBS, we found that when HCVcc was suspended in either plasma or PCs, the virus inactivation started after 27 J/cm^2^ of light exposure and complete inactivation (i.e., below the detection threshold) occurred following a 162 J/cm^2^ light dose in both plasma (Fig. 3A) and platelets (Fig. 3B). We found that suspension of HCVcc in either plasma or PCs (stored in plasma) alone (unexposed) resulted in a reduced infectivity relative to the predicted titer of 10^4^ ffu/mL. This is more obvious when PCs were used for HCVcc suspension (Fig. 3B). Taking this into account, the virucidal efficacy of 405 nm light is estimated to be ~ 3.8 log10 virus reduction relative to the spiked input virus (10^4^ ffu/mL) at a light dose of 162 J/cm^2^ in small volume samples (0.3-1.0 mL). Overall, at all the light exposure doses tested, we observed significant dose-dependent reductions in HCVcc titers in the exposed samples relative to the control (Fig. 3).

Analysis of the inactivation kinetics for HCVcc indicates that much of the inactivation occurs following the initial light exposure of 54 J/cm^2^, varying between plasma and PCs as illustrated in Fig. 4. For example, when the virus is resuspended in plasma, 78% of HCVcc was inactivated (Fig. 4A) after 54 J/cm^2^ exposure, but when the virus is suspended in PCs, a 27 J/cm^2^ light dose was sufficient to achieve ~ 80% of HCVcc inactivation (Fig. 4B).

### ROS-mediated inactivation of HCV

**Fig. 5:**
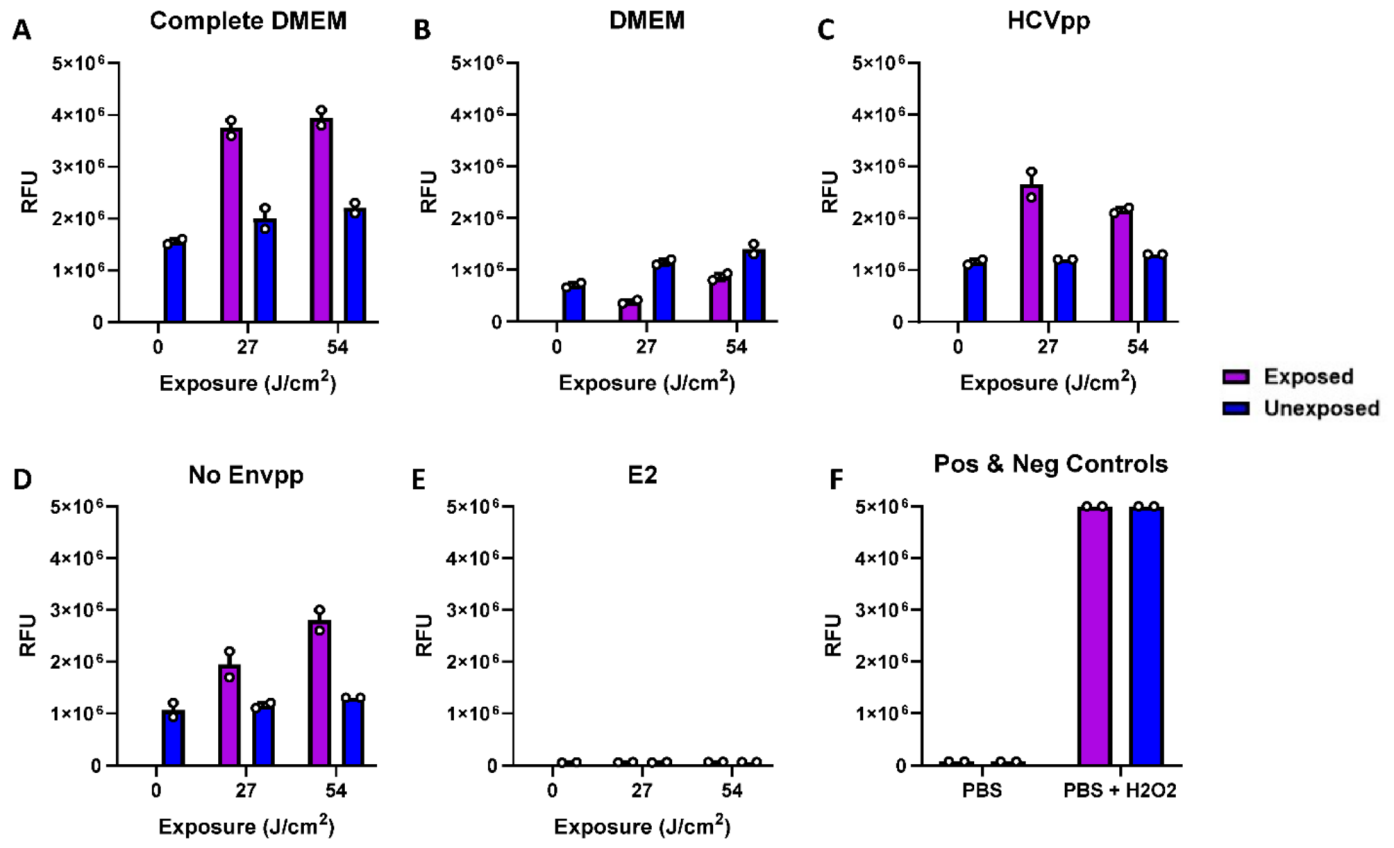
ROS detection in HCV pseudotyped particles. We assessed ROS production over time using a fluorescence indicator, H_2_DCFDA, in the following samples: (**A**) Complete DMEM (DMEM supplemented with 10% fetal bovine serum), (**B**) DMEM alone, (**C**) HCVpp (HCV pseudotyped particles in Complete DMEM; diluted 1:5 in PBS), (**D**) No Envpp (Non-enveloped control of HCVpp in Complete DMEM; diluted 1:5 in PBS), (**E**) E2 (purified HCV E2 glycoprotein in PBS), and (**F**) PBS (PBS alone served as a negative control and PBS + H_2_O_2_ serving as a positive control). Data is representative of 1 experimental replicate, where each sample was assessed in duplicate and assayed after 0, 27 and 54 J/cm^2^ light exposure. *RFU* relative fluorescence units.

We were intrigued by the inactivation of HCVcc in unmodified DMEM and PBS and sought to determine whether residual DMEM or residual complete DMEM containing fetal bovine serum could be a source of photosensitizers to facilitate ROS production. For this experiment, for biosafety reasons, we utilized pseudotyped virus particles HCVpp as a surrogate for HCVcc and non-enveloped pseudotyped particles, both of which were stored in complete DMEM, and soluble E2 protein in PBS. ROS detection was achieved using a fluorogenic compound, H_2_DCFDA (see Materials and Methods). In most of the samples studied, exposure to 405 nm light induced higher ROS as compared to unexposed control samples (Fig. 5). As seen in Fig. 5, when exposed, complete DMEM yielded higher amounts of ROS-mediated fluorescence after exposure to 27 J/cm^2^ of light (~ 4 × 10^6^ RFU; Fig. 5A), compared with DMEM alone after exposure to 27 J/cm^2^ light (mean 0.4 × 10^6^ RFU; Fig. 5B), which showed no increase in RFU between exposed and unexposed samples, suggesting that the carry-over of serum in the complete DMEM contributed to ROS induction. This rationale is further supported by the observation that HCVpp (~ 3 × 10^6^ RFU; Fig. 5C) and no Envpp (~ 2 × 10^6^ RFU; Fig. 5D), both of which contain residual amounts of complete DMEM, also yielded ROS-mediated fluorescence following exposure, whereas no ROS induction was detected in the sample containing the recombinant E2 protein that was not diluted in complete DMEM (Fig. 5E).

## Discussion

There have been several reports of visible violet-blue light-based viral inactivation in different scenarios^30^. Specific to the virucidal activity of 405 nm visible violet-blue light, we have previously shown complete inactivation of HIV-1 in human plasma and FCV in different biological matrices^8,9^, and others have shown reduction of SARS-CoV-2 and influenza A virus in PBS^31^. In this report we observed that suspension of HCVcc in either plasma or PCs (stored in plasma) alone resulted in non-light-induced slight loss of viral infectivity during incubation time. Given this observation, the virucidal efficacy of 405 nm light alone is estimated to be ~ 3.8 log10 virus reduction relative to the spiked input virus (10^4^ ffu/mL) at a light dose of 162 J/cm^2^ in small volume samples of all matrices tested. Furthermore, the viral inactivation kinetics data clearly show that the viral inactivation is 405 nm light dose dependent. Currently, the reason for this minor infectivity impediment with the plasma and PC matrices is not clear. This could be due to binding of the virus to plasma proteins or platelets, blocking of viral entry into Huh7.5 cells by the high numbers of cells present in the platelet samples, or the production of inhibitory cytokines by platelets. Additionally, platelets have been shown to possess antimicrobial and antiviral properties and release antimicrobial peptides (AMPs) into the surrounding biofluids (in this case plasma)^32^, which would lend support to the observed minor impediment to the infectivity observed in these matrices used in our studies.

With regards to the mechanism of pathogen inactivation by visible blue light (400–470 nm wavelength), a universally accepted hypothesis is that the light causes photoexcitation of photosensitizers such as porphyrins, flavins, and flavin derivatives present either within the microbe or, in the biological medium surrounding the microbe, resulting in the release of reactive oxygen species (ROS), which manifests microbicidal activity^29^. Recently we have demonstrated 405 nm light induced ROS-mediated inactivation of *Leishmania* parasite in plasma and platelets by direct measurement of ROS production^7^. Since bacteria and parasites are cellular organisms, it is conceivable that cellular photosensitizers will be the source of ROS induction upon 405 nm light exposure^29,33^. Virus particles, however, are acellular in nature and are not expected to contain photosensitizers within their particles, so while it is generally agreed that ROS induction is critical for viral inactivation, it has been suggested that there is a photosensitizer-independent mechanism of ROS induction^31^. However, a literature survey indicates that the majority of the studies utilized virus-infected cell culture supernatants as virus stocks; therefore, we hypothesized that the residual carryover culture media such as DMEM is sufficient to induce low levels of ROS upon light exposure even in PBS alone. However, if the culture media is complete, i.e., supplemented with bovine serum such as complete DMEM, the ROS induction is much higher. We tested this hypothesis as illustrated in Fig. 5 by direct measurement of ROS in different scenarios. Our results clearly supported the hypothesis and found that 405 nm light does not induce ROS in samples lacking residual amounts of DMEM. Further, the use of the HCV E2 alone confirmed that the hydrophobic nature or glycosylated form of the viral surface antigen is not sufficient to induce ROS in samples (as seen in Fig. 5E).

It is plausible that at least in the case of enveloped viruses like HCV (this report), and HIV-1^8^, the viral envelope constituents originating from the host cell membrane may have photosensitizer equivalents that could also augment ROS induction. Further studies in this direction are warranted.

There are certain limitations to these studies. We did not use full units of plasma and platelets. It is not possible to produce very high titer HCVcc viral stocks that can be used to spike large volumes of PCs and plasma. Therefore, small volumes of matrices were spiked to demonstrate the proof of concept that HCVcc can be inactivated in plasma and PC samples by 405 nm light treatment. We acknowledge that larger volume full units of plasma and PCs would require different conditions to be standardized such as depth of light penetration and light dosing. However, with regards to scalability and feasibility of this technology to larger volumes of plasma and PCs, it is worth mentioning that in our previous experiments with blood-borne bacteria where high titer bacteria can be grown easily, we showed that 405 nm light can effectively inactivate 99.9% of bacteria in ~ 300 mL of rabbit plasma in a bag at a 405 nm light dose of 144 J/cm^2^^34^ and 99.6–100% reduction of bacteria in ~ 200 mL of human PCs in a bag at a 405 nm light dose of 180 J/cm^2^^13^. These two reports illustrate that 405 nm light-based pathogen inactivation in large volumes of plasma and PCs in bags is feasible. We have previously reported other aspects that are relevant to the safety of a 405 nm light dose of 270 J/cm^2^ on platelet aggregation potential^6^ and platelet integrity relevant to the hemostatic potential of PCs, and potency of plasma coagulation factors (CFs)^15^. These studies demonstrated that the light-treatment had little to no effect on product functions relevant to hemostasis. Additionally, we have shown that platelet in vivo survival and recovery, which are the indicators of platelet integrity and quality, remain the same between 405 nm light-treated and untreated platelets in an immunodeficient murine model^13^. All these studies clearly provide a positive context to the feasibility and scalability of this 405 nm light-based pathogen reduction technology for the safety of plasma and PC units from infectious diseases. However, we acknowledge that further comprehensive studies are warranted on all these challenging aspects of the technology to assess its value as a novel PRT.

## Data Availability

All data generated or analyzed during this study are available from the corresponding author upon reasonable request.
